# Supplementary material for: Five Years Monitoring the Emergence of Unregulated Toxins in Shellfish in France (EMERGTOX 2018–2022)
Source: Mar Drugs. 2023 Jul 31;21(8):435. doi: 10.3390/md21080435 (PMC10456248; doi:10.3390/md21080435)
Supplement: Supplementary file 1 [file marinedrugs-21-00435-s001.zip › marinedrugs-2511406-supplementary.pdf]

Table S1. Recoveries of lipophilic toxins and domoic acid.

Toxin	Mean Recovery (%)
OA	99.0
DTX2	98.9
DTX1	98.9
YTX	85.2
homo YTX	82.9
AZA1	95.8
PTX2	96.7
SPX-13-desMe-C	92.0
PnTX G	94.3
PnTX A	93.3
GYM A	92.8
BTX3	94.1
PLTX	86.8
MC-RR	87.2
Nod-R	84.7
dmMC-RR	85.0
MC-LA	91.1
MC-LF	86.7
MC-LY	88.6
MC-LW	85.1
dmMC-LR	84.7
MC-LR	85.0
MC-YR	83.2
DA	81.5

Table S2. Detection and quantification limits (LOD, LOQ) of unregulated lipophilic toxins in shellfish.

**Lipophilic Toxins**	**LOD (μg/kg)**	**LOQ (μg/kg)**
PTXs, PnTXs (except PnTX-A)	1.2	3.5
PnTXA, GYMs	2.0	6.0
PLTX, OVTXs	150.0	450.0
BTXs	23.0	70.0
MCRR	1.3	4.0
NodR	1.5	4.5
dmMCRR	3.0	9.0
MCLA, MCLF, MCLY	2.7	8.0
MCLW	5.0	15.0
dmMCLR, MCLR, MCYR	10.0	30.0

Table S3: Recovery of Saxitoxin, Tetrodotoxin, Anatoxin and Cylindrospermopsin groups in Shellfish

**Toxins**	**Mean Recovery (%)**
STX	99.0
dc-STX	107.5
NEO	80.7
dc-NEO	89.5
TTX	93.3
C1	102.0
C2	91.5
GTX1	95.5
GTX2	100.0
GTX3	87.4
GTX4	91.8
GTX5	97.0
GTX6	85.0
dc-GTX2	100.0
dc-GTX3	92.0
ATX	94.0
CYN	95.8
do-CYN	72.3

Table S4. Detection and quantification limits (LOD, LOQ) of unregulated hydrophilic toxins in shellfish

**Hydrophylic Toxins**	**LOD (µg/kg)**	**LOQ (µg/Kg)**
TTX	11	30
ATX	8	17.5
CYN	2	5
doCYN	3	5

Table 5. Recovery of BMAA, DAB and AEG *in Shellfish*

**Toxin**	**Mean Recovery (%)**
BMAA	48
D3 BMAA	50
DAB	50
D5 DAB	48
AEG	61

Table S6. Elution gradients used for the three methods of lipophilic toxins and domoic acid analysis

OA, DTXs, YTXs		AZAs, PTXs, SPXs, PnTXs, GYMs, BTXs, PLTX/OVTX		DA, MCs, NOD	
Temps (min)	Proportion of mobile phase B (% B)	Temps (min)	% B	Temps (min)	% B
0.0	30%	0.0	30%	0.0	5%
1.0	70%	1.0	70%	1.0	50%
8.0	85%	10.0	95%	8.9	90%
9.5	100%	10.1	100%	9.0	100%
12.0	100%	12.0	100%	12.0	100%
12.1	30%	12.1	30%	12.1	5%
16.0	30%	16.0	30%	16.0	5%

Table S7. Mass spectrometer parameters applied according to the ionisation mode.

	OA, DTXs, YTXs Negative ionization	AZAs, PTXs, SPXs, PnTXs, GYMs, BTXs, PLTX, OVTXs Positive ionization	DA, MCs, NOD Positive ionization
Curtain gas (CUR)	20 psi	20 psi	20 psi
Collision Gas (CAD)	Medium	Medium	Medium
IonSpray Voltage (IS)	-4 500 V	5 500 V	5 500 V
Temperature (TEM)	500 °C	300 °C	550 °C
Ion Source Gas 1 (GS1)	40 psi	40 psi	40 psi
Ion Source Gas 2 (GS2)	60 psi	50 psi	55 psi
Interface Heater (ihe)	On	On	On

Table S8. Transitions for detection of unregulated lipophilic toxins and the source settings of mass spectrometer applied on the three sequence. Collision energy (CE), Declustering potential (DP), and collision cell exit potential (CXP)

Lipophilic toxin detected in positive ionization mode: PTXs, SPXs, PnTXs, GYMs, BTXs, PLTX

Compounds	Precursor ion (m/z)	Product ion [1] (m/z)	CE (eV)	DP (V)	CXP (V)
GYMA	508.3	392.3 (q)	49	111	9
	508.3	490.3 (Q)	34	111	11
GYMB	524.3	488.3 (q)	49	111	9
	524.3	506.3 (Q)	34	111	11
13-19-didesMeC	678.5	430.3 (q)	53	151	10
	678.5	164.2 (Q)	68	151	12
SPX-13-desMe-C	692.5	444.3 (q)	53	151	10
	692.5	164.2 (Q)	68	151	12
SPXdesMeD	694.5	444.3 (q)	53	151	10
	694.5	164.2 (Q)	68	151	12
PnTXG	694.5	458.3 (q)	53	151	10
	694.5	164.2 (Q)	68	151	12
PnTXA	712.5	458.3 (q)	53	151	10
	712.5	164.2 (Q)	68	151	12
PnTXF	766.5	488.3 (q)	53	151	10
	766.5	164.2 (Q)	68	151	12
PnTXE	784.5	488.3 (q)	53	151	10
	784.5	164.2 (Q)	68	151	12
PtTXABC	831.5	458.3 (q)	53	151	10
	831.5	164.2 (Q)	68	151	12
PLTX	1349.3	327.2 (q)	47	69	7
	900.0	327.2 (Q)	35	56	7
OVTXA	1333.3	327.2 (q)	47	69	7
	889.3	327.2 (Q)	35	56	7
	856.5	654.4 (Q)	67	136	17
PTX2	876.5	805.5 (q)	36	111	20
	876.5	823.5 (Q)	33	111	20
PTX1	892.5	821.5 (q)	36	111	20
	892.5	839.5 (Q)	33	111	20
PTX2sa et PTX2sa épi	894.5	805.5 (q)	36	111	20
	894.5	823.5 (Q)	33	111	20
PTX6	906.5	853.5 (q)	36	111	20
	906.5	871.5 (Q)	33	111	20
BTX2	912.5	319.3 (q)	37	81	7
	912.5	895.5 (Q)	19	81	22
BTX3	914.5	807.5 (q)	24	84	22
	914.5	725.5 (Q)	33	84	18

[1] Q: Quantitative transition; q: qualifier transition

Lipophilic cyanotoxins detected in positive ionization mode : Microcystins (MCs) and nodularin (Nod-R)

Compounds	Precursor ion (m/z)	Product ion [1] (m/z)	CE (eV)	DP (V)	CXP (V)
dmMCRR	512.8	103.1 (q)	88	98	6
	512.8	*135.1 (Q)*	*40*	*98*	*10*
MCRR	519.8	103.1 (q)	89	98	6
	519.8	*135.1 (Q)*	*42*	*98*	*10*
NodR	825.5	103.1 (q)	129	138	6
	825.5	*135.1 (Q)*	*79*	*138*	*10*
MCLA	910.5	135.1 (q)	81	146	9
	910.5	*776.5 (Q)*	*27*	*146*	*18*
dmMCLR	981.5	103.1 (q)	129	161	6
	981.5	*135.1 (Q)*	*99*	*161*	*10*
MCLF	986.5	135.1 (q)	88	154	9
	986.5	*852.5 (Q)*	*33*	*154*	*20*
MCLR	995.5	103.1 (q)	129	144	6
	995.5	*135.1 (Q)*	*95*	*144*	*10*
MCLY	1002.5	135.1 (q)	85	155	8
	1002.5	*868.5 (Q)*	*29*	*155*	*20*
MCLW	1025.5	375.3 (q)	54	156	10
	1025.5	*135.1 (Q)*	*95*	*156*	*10*
MCYR	1045.5	103.1 (q)	129	147	6
	1045.5	*135.1 (Q)*	*100*	*147*	*10*

[1] Q: Quantitative transition; q: qualifier transition

Table S9. Elution gradients used for the three methods of hydrophilic toxins analysis

PSTs and TTXs analysis (LC conditions A)			ATXs and CYNs (LC conditions B)		
Time (min)	Proportion of mobile phase B (% B)	Flow rate mL/min	Time (min)	% B	Flow rate mL/min
0.0	98%	0.4	0.0	98%	0.4
5.0	98%	0.4	5.0	98%	0.4
7.5	50%	0.4	11.5	50%	0.4
10.5	50%	0.4	13.0	50%	0.5
11.0	98%	0.4	13.5	98%	0.5
11.5	98%	08	14.0	98%	0.8
12.6	98%	0.8	14.6	98%	0.8
13.0	98%	0.4	15.0	98%	0.4

Table S10. Compound-dependent tandem mass spectrometry parameters for the unregulated hydrophilic toxins screened

Compounds	Ionisation mode	Precursor ion (m/z)	Product ion [1] (m/z)	CE (eV)	S-Lens (V)
TTX/4-epi-TTX	Positive	320.1	302.1(Q)	22	98
	Positive	320.1	162.1 (q)	36	98
5,6,11-trideoxy-TTX	Positive	272.1	254.1 (Q)	30	120
	Positive	272.1	162.1 (q)	35	120
4,9-anhydro-TTX	Positive	302.0	162.1 (Q)	32	147
	Positive	302.0	256.1 (q)	25	147
5-deoxy-TTX / 11-deoxy-TTX	Positive	304.1	286.1 (Q)	30	120
	Positive	304.1	176.0 (q)	30	120
ATX	Positive	166.1	131.1 (Q)	14	53
	Positive	166.1	105.1 (q)	16	53
HATX	Positive	180.1	145.1 (Q)	14	56
	Positive	180.1	117.1 (q)	20	56
CYN	Positive	416.2	336.1 (Q)	20	79
	Positive	416.2	194.1 (q)	34	79
doCYN	Positive	400.1	194.1 (Q)	32	99
	Positive	400.1	320.1 (q)	20	99

[1] Q: Quantitative transition; q: qualifier transition

Table S11. Source and MS parameters used for optimal detection of BMAA, DAB and internal standards.

Compounds	Precursor ion (m/z)	Product ion [1] (m/z)	CE (eV)	DP (V)	CXP (V)
BMAA	119	88 (q)	23	66	12
	119	44 (Q)	17	66	10
D_3BMAA	122	76 (q)	27	41	10
	122	47 (Q)	17	41	12
DAB	119	74 (q)	11	86	8
	119	101 (Q)	19	86	8
D_5DAB	124	78 (q)	19	131	10
	124	47 (Q)	13	131	4
AEG	119	102 (Q)	13	81	12

[1] Q: Quantitative transition; q: qualifier transition

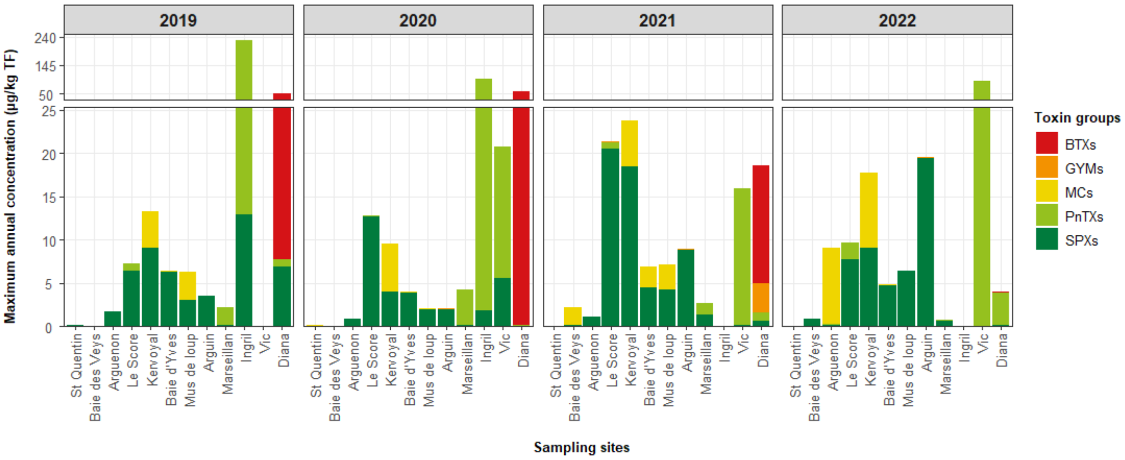

Fig. S1: maximum concentrations of unregulated toxins found in shellfish between 2019 and 2022 on the French coast
